# RARRES1 inhibits hepatocellular carcinoma progression and increases its sensitivity to lenvatinib through interaction with SPINK2

**DOI:** 10.1186/s13062-024-00459-0

**Published:** 2024-02-23

**Authors:** Yarong Guo, Bao Chai, Hezhao Zhang, Xinhao Chai, Yan Chen, Jun Xu, Liwei Qin, Yuting Chai

**Affiliations:** 1 Department of Digestive System Oncology, Shanxi Bethune Hospital, Shanxi Academy of Medical Sciences, Tongji Shanxi Hospital, Third Hospital of Shanxi Medical University, 030032 Taiyuan, Shanxi China; 2 Department of Oncology, The First Affiliated Hospital of Shanxi Medical University, 030001 Taiyuan, Shanxi China; 3 Department of Gastroenterology, Shanxi Bethune Hospital, Shanxi Academy of Medical Sciences, Tongji Shanxi Hospital, Third Hospital of Shanxi Medical University, 030032 Taiyuan, Shanxi China; 4 Department of Surgery, The First Affiliated Hospital of Shanxi Medical University, 030001 Taiyuan, Shanxi China; 5 Shanxi Medical University, 030001 Taiyuan, Shanxi China

**Keywords:** Lenvatinib, RARRES1, SPINK2, Sensitivity

## Abstract

**Background:**

Lenvatinib is an oral small molecule inhibitor approved for treating patients with unresectable hepatocellular carcinoma (HCC) worldwide. Increasing cell sensitivity to lenvatinib would be an effective method of improving therapeutic efficacy.

**Methods:**

High throughput methods was used to scan the differentially expressed genes (DEGs) related to lenvatinib sensitivity in HCC cells. Gain- and loss-function experiments were used to explore the functions of these DEGs in HCC and lenvatinib sensitivity. CO-IP assay and rescue experiments were utilized to investigate the mechanism.

**Results:**

We identified that RAR responder protein 1 (RARRES1), a podocyte-specific growth arrest gene, was among significantly upregulated DEGs in HCC cells following lenvatinib treatment. Functional analysis showed that ectopic RARRES1 expression decreased HCC progression in vitro and in vivo, as well as improving tumor sensitivity to lenvatinib, while RARRES1 silencing increased HCC cell proliferation and migration. Mechanistically, co-immunoprecipitation assays demonstrated that RARRES1 interacted with serine protease inhibitor Kazal-type 2 (SPINK2) in HCC cells. Further, SPINK2 overexpression suppressed HCC cell proliferation and migration, as well as increasing sensitivity to lenvatinib whereas SPINK2 knockdown promoted cell progression and decreased lenvatinib sensitivity. The mRNA and protein levels of RARRES1 and SPINK2 were low in HCC tissue samples, relative to those in normal liver tissue.

**Conclusions:**

Our findings highlighted that RARRES1 can inhibit HCC progression and regulate HCC sensitivity to lenvatinib by interacting SPINK2, representing a new tumor suppressor RARRES1/SPINK2 axis in HCC that modulates sensitivity to lenvatinib.

**Supplementary Information:**

The online version contains supplementary material available at 10.1186/s13062-024-00459-0.

## Background

Liver cancer is the sixth most common cancer worldwide, with 905,677 new cases in 2020, and the third highest cause of cancer-associated death globally [1]. Hence, liver cancer represents a worldwide health challenge, with an estimated incidence of > 1 million cases by 2025 [2]. As the most common form of liver cancer, hepatocellular carcinoma (HCC) accounts for approximately 90% of cases [2]. Diabetes, chronic alcohol consumption, obesity-associated nonalcoholic steatohepatitis, and infection by HBV or HCV are major risk factors for HCC [2]. Locoregional approaches, defined as imaging-guided liver tumor-directed procedures, play a leading part on the treatment of 50–60% of HCCs [3]. Radiofrequency is a standard method for local tumor ablation in early phase disease, while transarterial chemoembolization (TACE) is the typical treatment for intermediate-phase HCC [3, 4]. Overall survival rates are very poor in patients with advanced HCC and have not improved over the last decade, although some tyrosine kinase inhibitors (TKI) have been approved as first and second-line treatments [5].

Sorafenib has been the mainstay of HCC treatment for ten years, and newer modalities are not effective or do not confer any increase in therapeutic effect, until the emerging of lenvatinib [6]. As an oral small molecule TKi that inhibits various receptor tyrosine kinases, lenvatinib (Lenvima®) can be used to treat patients with unresectable HCC in the USA, EU, Japan, and China [7]. In intermediate-phase HCC patients with tumors exceeding the up-to-seven criteria, and for whom TACE is ineffective, lenvatinib can considerably increase overall survival (37.9 vs. 21.3 months) and progression-free survival (16.0 vs. 3.0 months) [8]. Further, lenvatinib plus pembrolizumab has potential antitumor activity in unresectable HCC [9].

Mechanistically, Jin et al. reported that the response of liver cancer to lenvatinib is limited by epithelial growth factor receptor (EGFR) activation, and that integrative therapy with the EGFR inhibitor, gefitinib, alongside lenvatinib may be of potential value for the approximately 50% of patients with advanced HCC who have high levels of EGFR [10]. Yi et al. revealed that lenvatinib reduced tumor programmed death ligand 1 levels and regulatory T cell differentiation, to enhance anti-PD-1 efficacy in patients with HCC by blocking FGFR4 [11]. Zheng et al. found that stomatin-like protein 2 (STOML2) could increase mitophagy by interacting with and stabilizing PINK1, which drives HCC metastasis and regulates the response of HCC to lenvatinib [12].

In this study, we scanned for genes related to lenvatinib sensitivity using high throughput methods to analyze Huh7 HCC cells treated with or without lenvatinib. We also conducted functional research to confirm the effects of identified genes in HCC. Our data reveal a new RARRES1 /SPINK2 axis with a tumor suppressor role in HCC, which decreased cell proliferation and migration and improve HCC cell sensitivity to lenvatinib.

## Results

### RNA seq-based identification of DEGs in lenvatinib-treated HCC cells

**Fig. 1:**
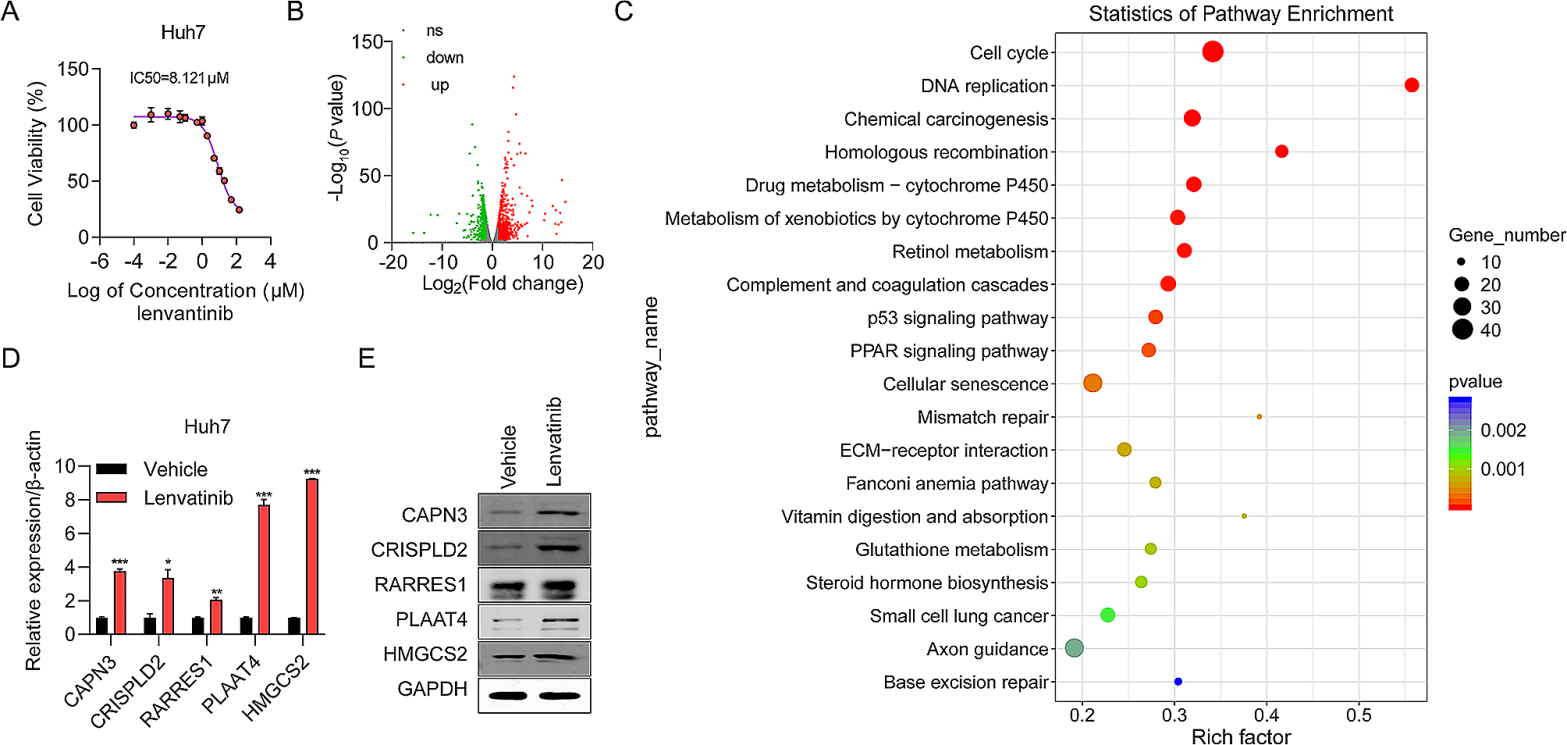
Upregulated genes in lenvatinib-treated HCC cells identified by RNA-seq analysis. **(A)** IC50 value of lenvatinib for HuH7 cells determined by CCK-8 assay. Each point on the dose–response curves represent three technical replicates. **(B)** Volcano map showing mRNAs upregulated (red) and downregulated (green) in the lenvatinib-treated group relative to the control group. **(C)** Top 20 enriched KEGG pathways in lenvatinib treated HCC cells. **(D)** Five new upregulated genes were verified by RT-qPCR analysis. **(E)** Five new upregulated genes were verified by western blot. *P < 0.05, **P < 0.01, ***P < 0.001

To determine the cytotoxicity of lenvatinib against HCC, we exposed Huh7 cells to various concentrations of lenvatinib for 48 h, and found that the IC50 value of lenvatinib was 8.121 µM (Fig. 1A). To identify targets of lenvatinib in HCC, the transcriptome of Huh7 cells exposed to Lenvatinib was examined by RNA-seq analysis. A total of 634 differentially expressed mRNAs were detected, including 343 upregulated and 291 downregulated genes. Significant DEGs were visualized as a volcano map (Fig. 1B). To further examine the effects of lenvatinib on HCC, we conducted KEGG enrichment analyses to identify possible lenvatinib-related pathways and biological functions. The most enriched KEGG terms were: cell cycle, DNA replication, chemical carcinogenesis, drug metabolism, and retinol metabolism (Fig. 1C). Furthermore, five genes (*CAPN3*, *CRISPLD2*, *RARRES1*, *PLAAT4*, and *HMGCS2*) were confirmed to be upregulated in lenvatinib-treated HCC cells by RT-qPCR and western blot assays (Fig. 1D-E). These genes may significantly influence HCC cell sensitivity to lenvatinib.

### RARRES1 is downregulated in HCC tissues and suppression of RARRES1 decreases cell sensitivity to lenvatinib

**Fig. 2:**
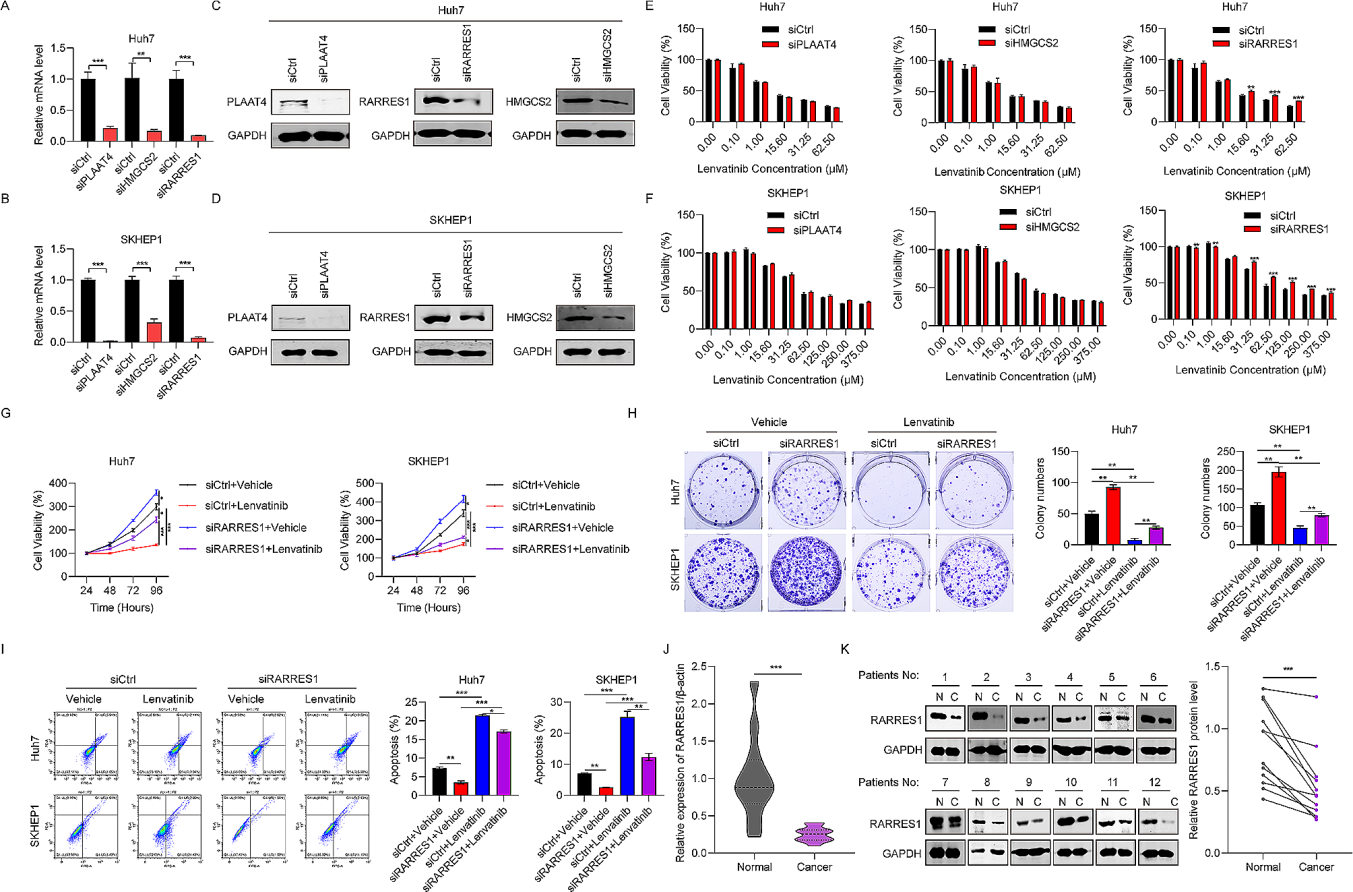
RARRES1 was downregulated in HCC tissues and suppressing RARRES1 decreased HCC cell sensitivity to lenvatinib. **(A-D)** RT-qPCR and western blot assays were used to detect the knockdown efficiency of three genes in Huh7 and SKHEP1 cells. **(E–F)** Sensitivity to lenvatinib in siPLAAT4/siRARRES1/siHMGCS2-treated Huh7 and SKHEP1 cells, determined by CCK-8 assay. **(G)** HuH7 and SKHEP1 cells were treated with siCtrl + vehicle, siRARRES1 + vehicle, siCtrl + lenvatinib, and siRARRES1 + lenvatinib for 24, 48, 72 and 96 h and cell viability determined by CCK-8 assay. **(H)** Results of cell colony formation assay. **(I)** HuH7 and SKHEP1 cells apoptosis after treatment with siRARRES1 and/or lenvatinib, determined by flow cytometry. **(J–K)** RT-qPCR and western blot assays were used to examine RARRES1 mRNA and protein levels in 12 matched HCC and adjacent normal liver tissue samples. *P < 0.05, **P < 0.01, ***P < 0.001

To further evaluate the function of the five genes confirmed to be upregulated in response to lenvatinib (*CAPN3*, *PLAAT4*, *RARRES1*, *HMGCS2*, and *CRISPLD2*), we knocked down the expression of each in Huh7 and SKHEP1 using siRNAs. The results of RT-qPCR showed that siRNAs targeting *PLAAT4*, *RARRES1*, and *HMGCS2* were effective (Fig. 2A-B). Western blot analysis also confirmed the knockdown efficiency of these three genes (Fig. 2C-D). To assess the possible effects of knockdown of these genes on HCC cell sensitivity to lenvatinib, Huh7 and SKHEP1 cells transfected with siRNAs were treated with a series of lenvatinib concentrations. The results showed that siRARRES1 could decrease Huh7 cell sensitivity to lenvatinib at concentrations of 15.6, 31.25, and 62.5 µM (Fig. 2E); similar results were obtained using SKHEP1 cells (Fig. 2F). Therefore, we chose to conduct further analysis of the *RARRES1* gene. CCK-8 assays were adopted to determine the viabilities of Huh7 and SKHEP1 cells treated with siRARRES1 and/or lenvatinib (31.25 µM) for 24, 48, 72, and 96 h. Lenvatinib treatment markedly reduced cell viability of the two HCC cell lines compared to vehicle-treated control cells, while RARRES1 knockdown greatly increased cell viability. Further, combination treatment with siRARRES1 and lenvatinib led to a higher HCC cell proliferation rate than treatment with lenvatinib alone, indicating that inhibition of RARRES1 decreased HCC cell sensitivity to lenvatinib (Fig. 2G). Similarly, more colonies were formed in the siRARRES1 + lenvatinib group relative to the lenvatinib group in cell colony forming assays, verifying that RARRES1 knockdown decreased HCC cell sensitivity to lenvatinib (Fig. 2H). Levels of Huh7 and SKHEP1 cell apoptosis on treatment with siRARRES1 and/or lenvatinib were examined by flow cytometry. The results demonstrated that lenvatinib alone significantly triggered apoptosis of Huh7 and SKHEP1 cells, whereas RARRES1 knockdown reduced rates of apoptosis (Fig. 2I). Finally, RT-qPCR and western blot analyses were conducted to evaluate RARRES1 expression in 12 pairs of HCC samples. HCC tissue samples exhibited much lower expression of RARRES1 than normal liver tissues at both the mRNA and protein levels (Fig. 2J–K). These data show that suppression of RARRES1 decreases HCC cell sensitivity to lenvatinib, and that RARRES1 is expressed at low levels in HCC tissue.

### Ectopic expression of RARRES1 increases HCC cell sensitivity to lenvatinib

**Fig. 3:**
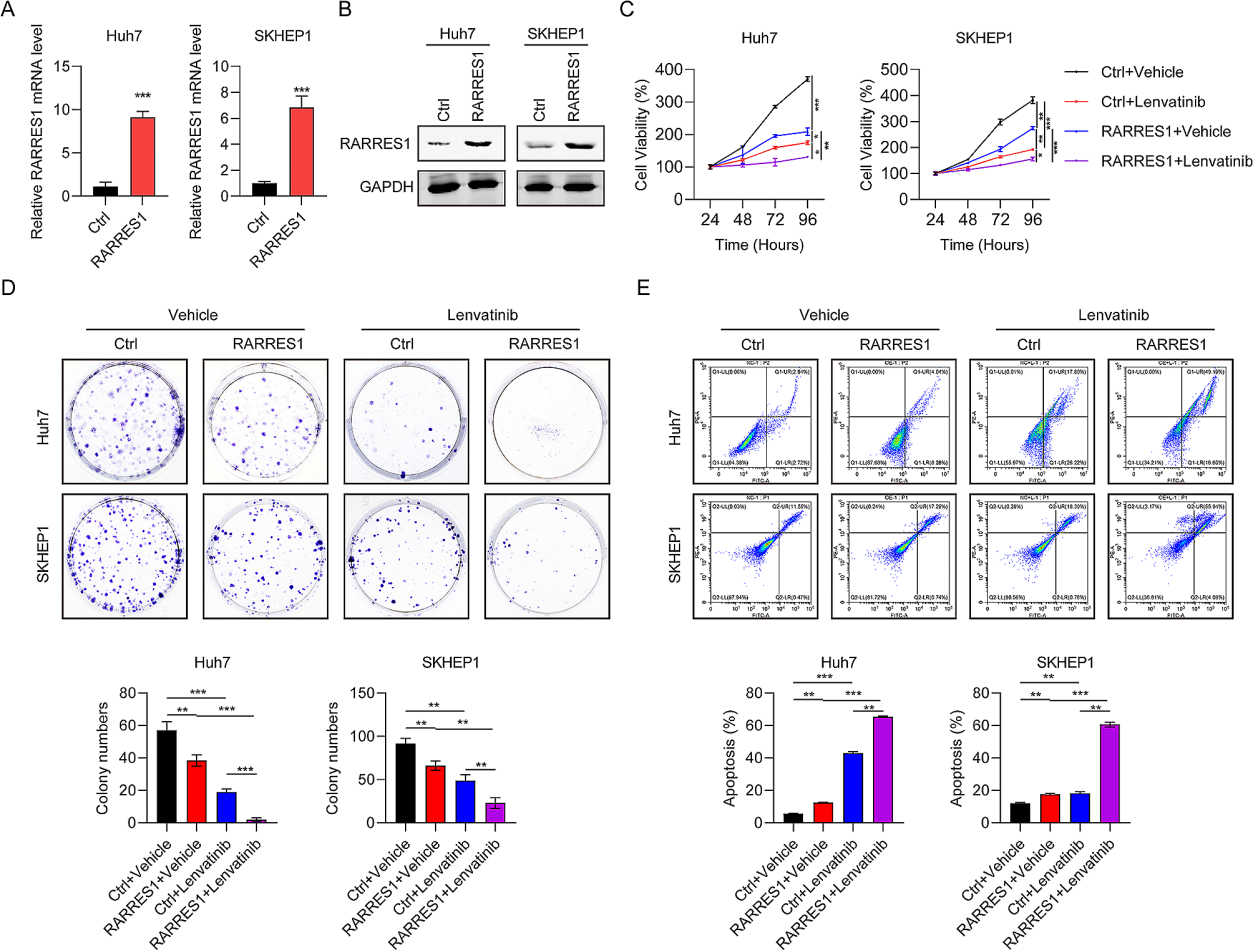
Ectopic expression of RARRES1 increased cell sensitivity to lenvatinib. **(A–B)** RT-qPCR and western blot were used to examine RARRES1 overexpression in Huh7 and SKHEP1 cells. **(C-E)** The effects of RARRES1 on in vitro cell proliferation and apoptosis of Huh7 and SKHEP1 cells determined by **(C)** CCK-8 assay, **(D)** colony formation assay, and **(E)** apoptosis assay. *P < 0.05, **P < 0.01, ***P < 0.001

Our data showed that RARRES1 was expressed at low levels in HCC tissues, therefore we evaluated the function of RARRES1 in HCC cells and its role in cell sensitivity to lenvatinib. Plasmids for RARRES1 overexpression were transfected into Huh7 and SKHEP1 cells and the efficiency of RARRES1 overexpression confirmed by RT-qPCR and western blot (Fig. 3A-B). Ectopic RARRES1 expression in Huh7 and SKHEP1 cells resulted in a significant decrease of cell viability (Fig. 3C) and colony formation (Fig. 3D), as well as increasing the sensitivity of HCC cells to lenvatinib. Further, the percentage of apoptotic cells was clearly increased following RARRES1 overexpression relative to the control group, while lenvatinib treatment further increased the apoptosis rate (Fig. 3E). These data show that RARRES1 overexpression restrained cell proliferation, induced apoptosis, and enhanced sensitivity to lenvatinib.

### The roles of RARRES1 in HCC cell migration and epithelial-mesenchymal transition (EMT)

**Fig. 4:**
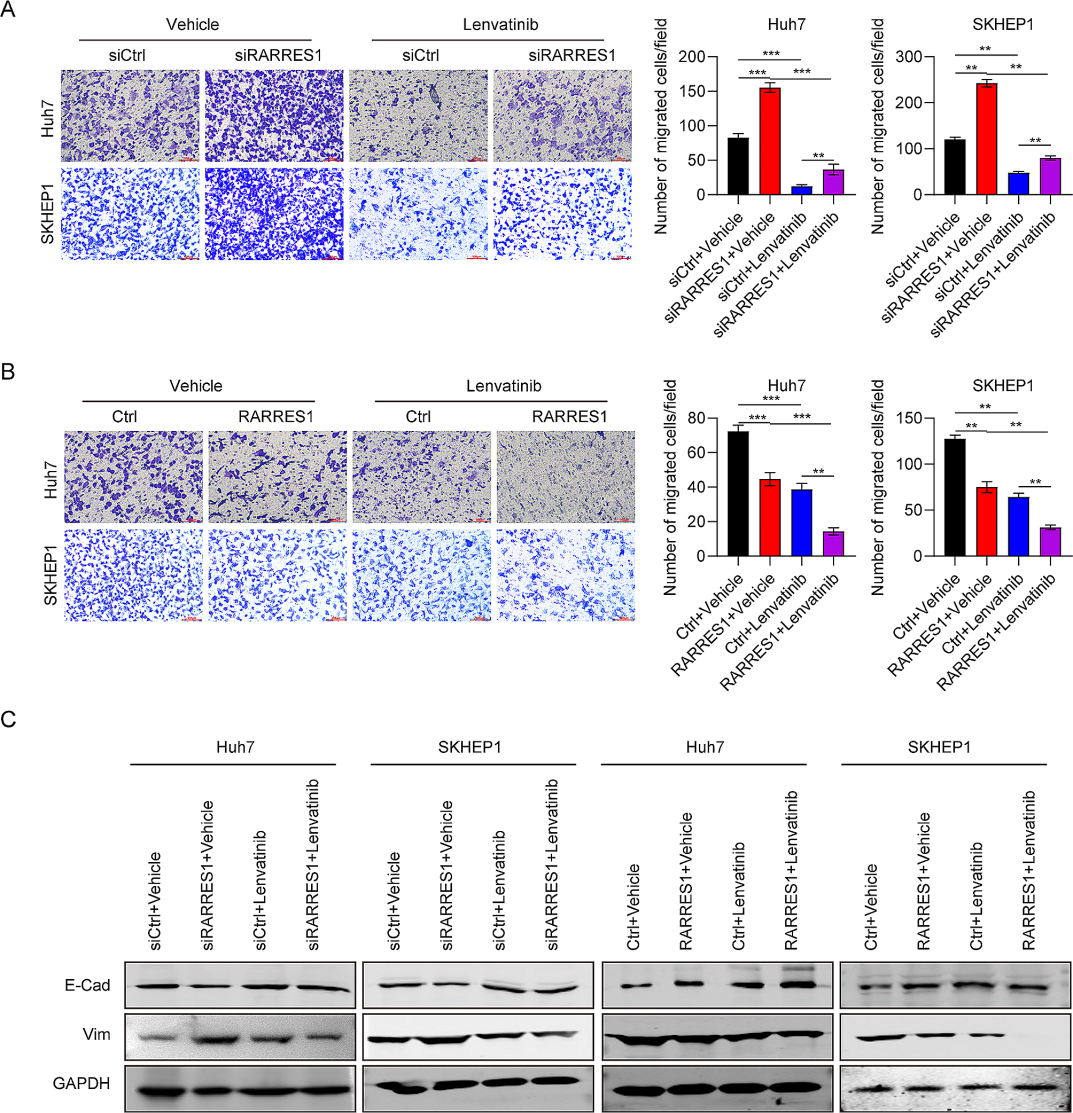
Regulation of RARRES1 expression influenced cell migration and EMT with or without lenvatinib treatment. **(A)** Representative images and statistical analysis of data from transwell assays in Huh7 and SKHEP1 cells transfected with siCtrl, siRARRES1, siCtrl + lenvatinib, and siRARRES1 + lenvatinib. **(B)** Representative images and statistical analysis of data from transwell assays in Huh7 and SKHEP1 cells transfected with Ctrl, RARRES1, Ctrl + lenvatinib, and RARRES1 + lenvatinib. **(C)** Protein expression of EMT markers (E-Cadherin and Vimentin) in Huh7 and SKHEP1 cells transfected with siCtrl, siRARRES1, siCtrl + lenvatinib, and siRARRES1 + lenvatinib, or Ctrl, RARRES1, Ctrl + lenvatinib, and RARRES1 + lenvatinib. **P < 0.01, ***P < 0.001

Next, we evaluated the roles of RARRES1 in HCC cell migration and EMT under lenvatinib treatment. Silencing RARRES1 significantly increased migrated cell numbers and decreased Huh7 and SKHEP1 cell sensitivity to lenvatinib (Fig. 4A). Conversely, ectopic RARRES1 expression inhibited Huh7 and SKHEP1 cell migration by 30% and 50%, respectively, compared with control cells (Fig. 4B). Further, there were fewer migrated cells in the RARRES1 + lenvatinib group than in cells treated with lenvatinib alone, indicating that RARRES1 overexpression promoted HCC cell sensitivity to lenvatinib (Fig. 4B). Regarding EMT markers, E-Cadherin levels were markedly reduced in Huh7 and SKHEP1 cells after RARRES1 knockdown, while those of Vimentin were clearly increased (Fig. 4C). E-Cadherin expression was upregulated compared with controls in response to both RARRES1 overexpression and lenvatinib treatment, and levels were highest in the RARRES1 + lenvatinib group, confirming the positive effect of RARRES1 on lenvatinib sensitivity (Fig. 4C). In conclusion, these data show that RARRES1 exerts a tumor suppressor effect on HCC cell migration and EMT, and high RARRES1 expression acts synergistically with lenvatinib in HCC.

### RARRES1 interacts with SPINK2

**Fig. 5:**
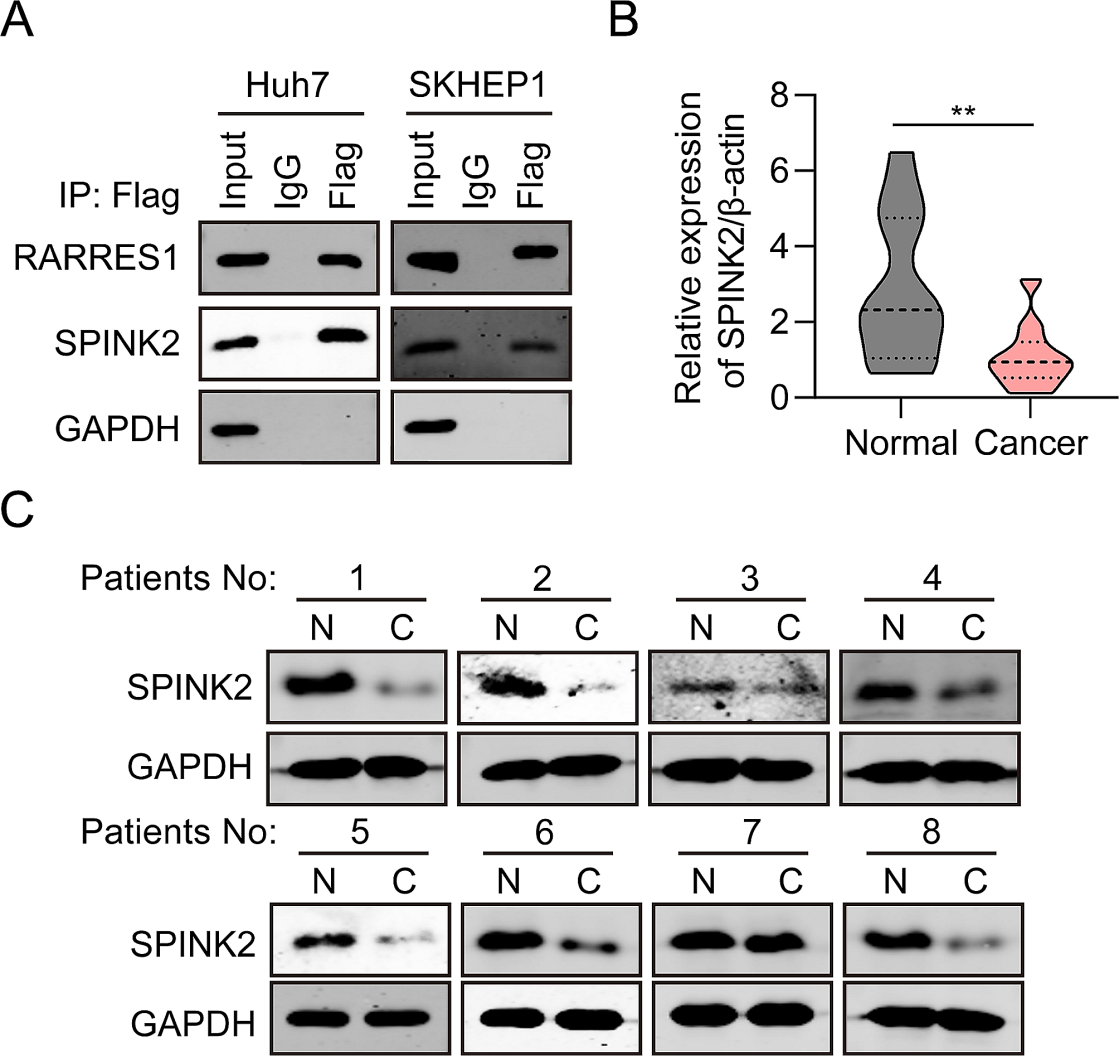
RARRES1 interacts with SPINK2. **(A)** Interaction between RARRES1 and SPINK2 in Huh7 and SKHEP1 cells was explored by immunoprecipitation with anti-SPINK2 or anti-RARRES1 antibodies, followed by western blot analysis. **(B–C)** RT-qPCR and western blot examination of SPINK2 expression in 12 pairs of HCC and normal liver tissue samples

It has been reported that RARRES1 could interacts with SPINK2 to inhibit cellular invasion of testicular carcinoma cells [13]. To determine the biological function of RARRES1 in HCC depend on binding proteins, CO-IP assays were conducted to determine whether RARRES1 and SPINK2 physically interact in Huh7 and SKHEP1 cells. Vectors for expression of recombinant Flag-tagged RARRES1 protein were constructed, to further evaluate the relationship between RARRES1 and SPINK2. CO-IP assays were conducted to determine whether RARRES1 and SPINK2 physically interact in Huh7 and SKHEP1 cells. Interaction of endogenous RARRES1 with SPINK2 in Huh7 and SKHEP1 cells was also confirmed by reciprocal CO-IP assay (Fig. 5A). We next analyzed the expression of SPINK2 in 12 pairs of clinical HCC tissues samples. RT-qPCR and western blot both demonstrated that SPINK2 mRNA and protein levels were markedly lower in HCC tumor samples than those in normal liver tissue (Fig. 5B-C). These results indicate that SPINK2 may be involved in HCC progression.

### The role of SPINK2 in HCC cell proliferation and migration

**Fig. 6:**
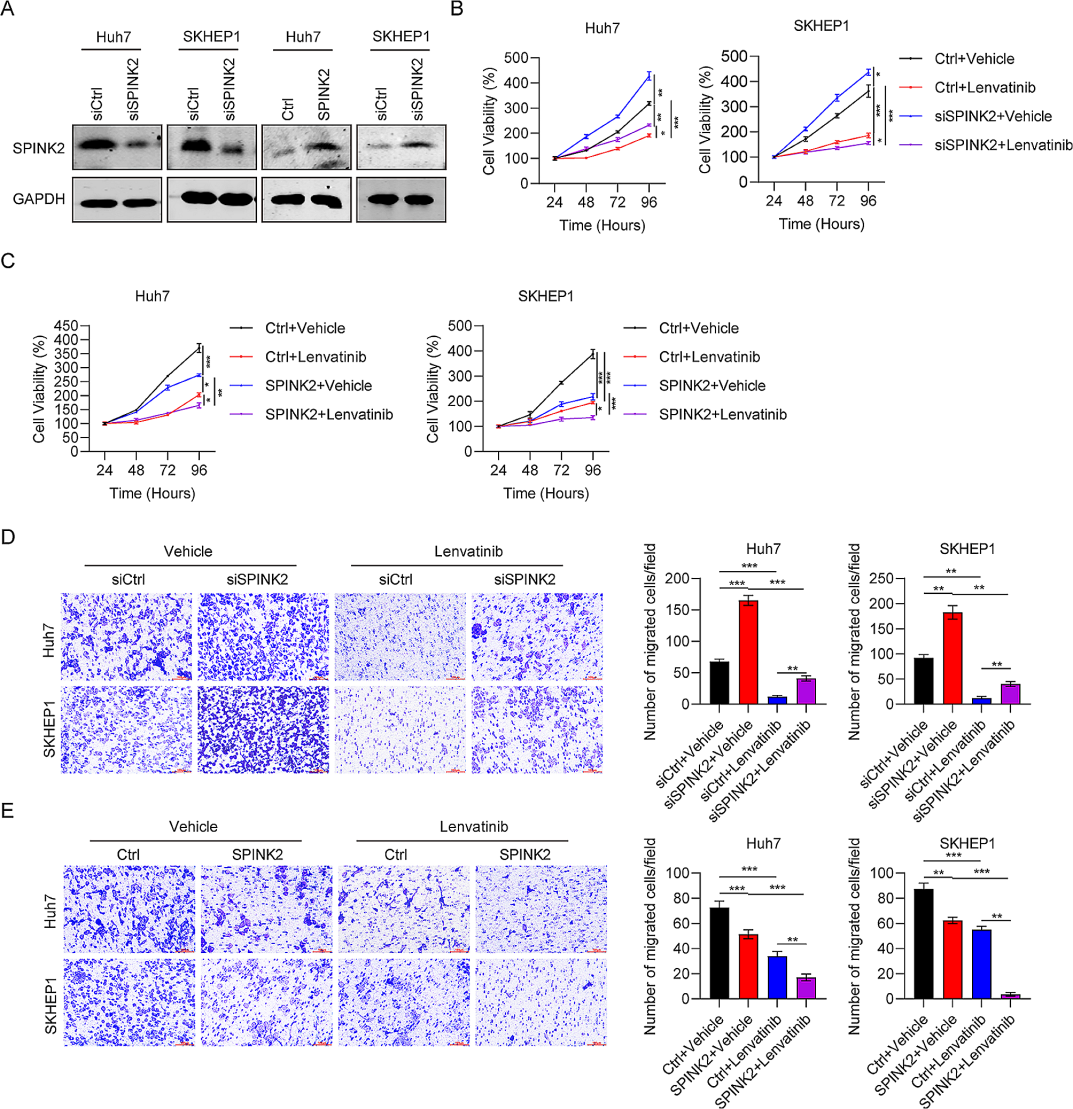
SPINK2 regulates HCC cell migration and EMT. **(A)** SPINK2 levels in Huh7 and SKHEP1 cells transfected with siSPINK2 or siCtrl detected by western blot. (B-E) The role of SPINK2 in HCC cell propagation, migration, and sensitivity to lenvatinib using **(B-C)** CCK-8 and **(D-E)** transwell assays. *P < 0.05, **P < 0.01, ***P < 0.001

To examine the effect of SPINK2 on HCC, we knocked down or overexpressed SPINK2 in Huh7 and SKHEP1 cells. The knockdown efficiency of SPINK2-specific siRNA and overexpression efficiency of SPINK2 plasmid were confirmed by western blot (Fig. 6A). Then, the roles of SPINK2 in HCC cell proliferation and migration were examined using CCK-8 and transwell assays, respectively. We found that SPINK2 silencing increased cell viability and migration of Huh7 and SKHEP1 cells, whereas ectopic expression of SPINK2 decreased cell proliferation and migration (Fig. 6B-E). Furthermore, Huh7 and SKHEP1 cells with SPINK2 knocked down became more resistant to lenvatinib, while SPINK2 overexpression sensitized Huh7 and SKHEP1 cells to lenvatinib (Fig. 6B-E). In conclusion, these findings show that HCC cells expressing high levels of SPINK2 were more sensitive to lenvatinib.

### RARRES1 suppresses tumor growth and increases sensitivity to lenvatinib in vivo

**Fig. 7:**
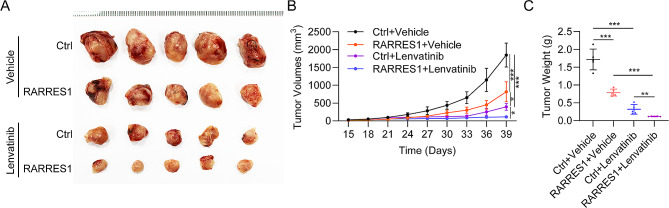
RARRES1 suppressed tumor growth in vivo and increased sensitivity to lenvatinib. **(A)** Tumor sizes in four groups of Huh7 xenografts treated with Ctrl, RARRES1, lenvatinib, and RARRES1 + lenvatinib. **(B)** The tumor growth curves were generated for each group by measuring tumor volumes every three days. **(C)** Weights of tumors dissected from mice in the four groups. *P < 0.05, ***P < 0.001

The anti-tumor activity of RARRES1 and lenvatinib against HCC was next examined in vivo. As shown in Fig. 7A, RARRES1 overexpression or lenvatinib treatment alone both exhibited anti-tumor effects in Huh7 xenografts. Importantly, RARRES1 overexpression combined with lenvatinib could significantly reduce tumor size than RARRES1 overexpression or Lenvatinib alone. Analysis of tumor growth curves and tumor weight also demonstrated that both RARRES1 upregulation and treatment with lenvatinib resulted in smaller tumors relative to the control group, while mice with tumors with RARRES1 upregulation that were also treated with lenvatinib had the smallest tumors among the four groups (Fig. 7B-C). These results indicate that RARRES1 overexpression improves tumor cell sensitivity to lenvatinib.

## Discussion

Lenvatinib, an oral multi-target TKI, is the standard-of-care, either as a monotherapy or in combination with other anticancer agents, for treatment of radioiodine-refractory differentiated thyroid carcinoma, renal cell carcinoma, hepatocellular carcinoma, and endometrial carcinoma, and is being evaluated for use in therapy for some other types of tumors [14]. There is evidence from preclinical research that lenvatinib has anti-tumorigenesis and immunomodulatory activity [15]. Given its non-inferior effectiveness to sorafenib and manageable tolerability profile, lenvatinib represents a long-awaited alternative option to sorafenib for systematic treatment of patients with unresectable HCC [7]. Therefore, exploring the functional mechanism of lenvatinib and searching for genes that act synergistically with lenvatinib may generate new HCC therapy approaches.

In this study, we used RNA-seq to scan for the DEGs between lenvatinib-treated and -untreated HCC cells and identified 634 DEGs (343 upregulated and 291 downregulated). KEGG enrichment analyses revealed that pathways involving the cell cycle, apoptosis, and changes in metabolism, among others, were the most enriched terms. Further, we selected a number of new genes that were previously unreported in HCC, but were expressed at higher levels in response to lenvatinib treatment, and verified these findings by RT-qPCR. Western blots were also conducted to confirm the expression of these molecules, and only five proteins (CAPN3, CRISPLD2, RARRES1, PLAAT4, and HMGCS2) were shown to be upregulated. We also synthesized siRNAs targeting *CAPN3*, *CRISPLD2*, *RARRES1*, *PLAAT4*, and *HMGCS2*, to knockdown these five genes in HCC cells; however, only three (*RARRES1*, *PLAAT4*, and *HMGCS2*) were effectively knocked down. Next, we tested lenvatinib sensitivity in two HCC cell lines (Huh7 and SKHEP1), and found that only RARRES1 knockdown could decrease cell sensitivity to lenvatinib at concentrations of 31.25 and 62.5 µM; therefore, further analysis of RARRES1 was conducted.

RAR responder protein 1 (RARRES1), a podocyte-specific growth arrest gene, is controlled by high doses of both retinoid acid and TNF-alpha, and contributes to maintenance of podocytes in a quiescent state [16]. RARRES1 has a tumor suppressor role in numerous human cancers, including breast cancer [17], prostate cancer [18], osteosarcoma [19], choriocarcinoma [20], and kidney renal clear cell carcinoma [21]. RARRES1 is enriched in glioblastoma multiforme (GBM), particularly WHO grade-IV cases; and high expression of RARRES1 is a predictor of poor prognosis, indicating that it may participate in GBM pathogenesis, and is a potential therapeutic target in this context [22]. We used siRNAs to knockdown RARRES1 and determine the role of RARRES1 in liver cancer cell proliferation and apoptosis, and the results showed that silencing RARRES1 increased cell viability and cell colony numbers, as well as decreasing the rate of apoptosis and HCC cell sensitivity to lenvatinib. Conversely, RARRES1 overexpression promoted lenvatinib cell sensitivity. Transwell assays and evaluation of EMT markers also demonstrated the tumor suppressor effect of RARRES1 on HCC cell lines, and its positive role in enhancing cell sensitivity to lenvatinib. The anti-tumor activity of RARRES1 in vivo was examined using a tumor xenograft model, and the results demonstrated that RARRES1 overexpression improved tumor cell sensitivity to lenvatinib, with smaller tumor size and weight observed in the RARRES1 + lenvatinib group, relative to treatment with RARRES1 or lenvatinib alone. Moreover, we also found low levels of RARRES1 expression in clinical tissue samples from patients with HCC patients. Mechanistically, we detected interaction between RARRES1 and SPINK2 using a CO-IP assay.

The serine protease inhibitor Kazal type (SPINK) family is the largest branch of the serine protease inhibitor family, comprising SPINK1–14. SPINKs exert significant effects on sperm maturation and capacitation, pancreatic physiology and disease, Nager syndrome, inflammation, and the skin barrier [23]. SPINK protein family members have a Kazal domain at the C-terminus, which is a protein domain largely found in serine proteases [24]. There is a close relationship between the expression of SPINK2 and cancer growth, and high levels of SPINK2 transcript are detected in patients with primary skin follicular center cell lymphoma [25]. SPINK2 levels are greatly increased in most of the leukemia cell lines that have been examined and this protein exerts a significant effect on tumor progression and response to treatment [26]. On downregulation of SPINK2 in testicular cancer tissues, the combined effect of SPINK2 and RARRES1 significantly inhibits testicular cancer cell EMT by downregulating the uPA/uPA receptor signaling pathway [13]. In this study, SPINK2 mRNA and protein expression were downregulated in clinical tissues from patients with HCC relative to normal liver tissue. SPINK2 overexpression decreased HCC cell proliferation and migration and promoted cell sensitivity to lenvatinib, while SPINK2 knockdown had the opposite effects.

## Conclusions

In conclusion, RARRES1 expression is strongly associated with HCC cell proliferation, migration, and apoptosis. In vitro and in vivo functional studies validated the antitumor effects of RARRES1 and its role in increasing HCC cell sensitivity to lenvatinib through promotion of SPINK2 expression. This research provides insights that could inform follow-up investigation into HCC molecular pathogenesis.

### Methods

#### Clinical patients

Total of twelve pairs of patients with HCC were used to analyze RARRES1 and SPINK2 expression at the mRNA and protein levels. 12 pairs of fresh human HCC samples and normal tissues were collected from The First Affiliated Hospital of Shanxi Medical University. All patients received neither chemotherapy nor radiotherapy prior to surgery. All of the HCC bioptic specimens from resected HCC and then were maintained in -80 °C. The clinical characteristics of the HCC examined in the Supplementary Table 1. The ethics committee of The First Affiliated Hospital of Shanxi Medical University approved this research. Written consent was signed by all patients.

### Cell culture and cell transfection

Human liver cancer cell lines (Huh7 and SKHEP1) were from the Shanghai Cell Bank of the Chinese Academy of Sciences (Shanghai, China) and were cultivated in DMEM with 10% FBS, glutamine, and 1% penicillin-streptomycin (Gibco) at 37 °C and in 5% CO_2_. Lenvatinib was purchased from APExBIO chemicals.

Four siRNAs each targeting to *PLAAT*, *RARRES1*, *HMGCS2*, and *SPINK2*, separately, and a corresponding control siRNA were purchased from Riobio corporation (Guangzhou, China). Plasmids for overexpression of RARRES1, and SPINK2 were constructed using the pcDNA3.1 vector. Lipofectamine 2000 (Invitrogen) was used to transfect plasmid constructs and oligonucleotides into HCC cells, based on the product instructions.

### RNA sequencing

Huh7 cells were treated with lenvatinib for 48 h; control group cells were treated with DMSO. RNA sequencing analysis was performed by KangChen Bio-Tech (China). Differentially expressed genes (DEGs) between the lenvatinib and control groups were subjected to KEGG pathway analysis. Molecular mechanisms potentially influenced by lenvatinib were determined by gene set enrichment analysis. All the analyses were carried on using the R package, ClusterProfiler. The expression of these DEGs previously un-reported in HCC were confirmed by quantitative real-time PCR (RT-qPCR) and western blot.

### RT-qPCR

Total RNA samples were extracted using Trizol (Invitrogen), and M-MLV reverse transcriptase (Invitrogen) and oligo (dT)12–18 applied to prepare cDNAs. RT-qPCR was conducted in triplicate in a thermal cycler, with 20 µl reaction mixtures containing 10 µl Fast SYBR Green Master Mix, 50 ng cDNAs, and gene-specific forward and reverse primers (1 µM final concentration). PCR primers are presented in Table 1. Relative expression levels of target cDNAs were calculated after normalization of relative intensity of target cDNA to that of β-actin.

**Table 1:** Primers used in this research

Gene	Gene ID	Forward sequence 5’-3’	Reverse sequence 5’-3’
*RARRES1*	5918	AAACCCCTTGGAAATAGTCAGC	GGAAAGCCAAATCCCAGATGAG
*SPINK2*	6691	TCTCTGATCCCTCAATTTGGTCT	CCACACACAGGGTTAAAGTGTC
*CAPN3*	825	GTCCTTAACACAGTCGTGAACA	TGAGCGCAATCATGCTACGG
*CRISPLD2*	83,716	GCCCAACGTCACTCTCTTAGA	GTTGTGCAGCATGAGGATCTC
*HMGCS2*	3158	GACTCCAGTGAAGCGCATTCT	CTGGGAAGTAGACCTCCAGG
*PLAAT4*	5920	GAGATTTTCCGCCTTGGCTAT	CCGGGGTACTCACTTGGAG
*β-actin*	60	CATGTACGTTGCTATCCAGGC	CTCCTTAATGTCACGCACGAT

### Western blot

Protein samples were separated by 12% SDS-PAGE and transferred to polyvinylidene fluoride membranes. After blocking, membranes were incubated with primary antibodies for 12 h at 4 °C, followed by incubation with horseradish peroxidase-conjugated antibodies at room temperature for 1 h. Labeled antibodies were detected using an ECL kit (Amersham, UK). The antibodies used in this research were: anti- RARRES1 (ab87115, Abcam), anti-SPINK2 (PA5-72754, Thermo Fisher Scientific, USA), anti-CAPN3 (HPA040052, Sigma-Aldrich), anti-CRISPLD2 (PA5-110992, Thermo Fisher Scientific), anti-PLAAT4 (ab96468, Abcam), anti-HMGCS2 (ab157225, Abcam), anti-E-cad (ab40772, Abcam), anti-Vim (ab92547, Abcam), and anti-GAPDH (Cat: #2118S, Cell Signaling Technology, USA).

### Cell viability determination

To determine the toxicity of lenvatinib to HCC, Huh7 cells were treated with various concentrations of lenvatinib for 48 h, and a Cell Counting Kit-8 (CCK-8) assay used to measure cell viability. IC50 values were detected by GraphPad Prism 8.0 using a 3-parameter dose–response model.

To determine the effects of targeted gene knockdown on cell sensitivity to lenvatinib, cells transfected with the indicated siRNAs were seeded in 96-well plates at 3,000 cells per well, followed by overnight incubation. The next day, after rinsing cells, fresh medium containing DMSO or lenvatinib (0.1–375 µM) was added for 72 h. CCK-8 assays were conducted to measure cell viability and IC50 values calculated.

To determine the effects of RARRES1 and SPINK2 expression on cell sensitivity to lenvatinib, cells transfected with the indicated siRNAs or plasmids were seeded, incubated, rinsed, and treated with lenvatinib for 24, 48, 72 and 96 h. Cell viability was measured using CCK-8 assays.

### Cell colony formation assay

HCC cells (*n* = 800) transfected with the indicated plasmids or siRNAs for 24 h were seeded in 6-well plates and cultured in DMEM medium supplemented with 10% FBS for 14 days. Then, cells were fixed with methanol, stained with crystal violet solution, and colonies containing ≥ 30 cells counted under a microscope.

### Flow cytometry to detect apoptosis

Annexin V-FITC and propidium iodide (PI) were used to detect apoptosis by flow cytometry. Briefly, cells transfected with siRARRES1 were cultured in 12-well plates, followed by treatment with 20 µmol/L lenvatinib for 48 h. Cells were then incubated with Annexin V-FITC and PI for 20 min, followed by flow cytometry.

### Transwell assay

Transwell assays were conducted to assess cell migration ability. Briefly, cells were seeded into 6-well plates and incubated overnight, followed by 24-h transfection with the indicated siRNAs and/or lenvatinib (20 µmol/L) treatment. Next, cells were collected and reseeded in serum-free DMEM at 2 × 10^4^ cells per well in upper transwell inserts with 8 μm pores. DMEM containing 20% FBS was added to the lower wells as a chemoattractant. After incubation for 24 h, cells were fixed in methanol (10 min, room temperature), followed by immediate transfer to − 20 °C for overnight incubation. Cells were then stained with 50 µg/mL PI for 30 min, washed twice with PBS, and examined using a Nikon ECLIPSE 80i microscope to determine the number of cells on each transwell membrane.

### Co-immunoprecipitation (CO-IP)

Huh7 and SKHEP1 cells were transfected with the indicated plasmids for 24 h. IP buffer supplemented with protease inhibitor cocktail and phosphatase inhibitor was used to lyse cells. After overnight incubation with the indicated antibodies at 4 °C, cell lysates were incubated with 20 µl of Protein G plus/Protein A-agarose for 2 h at 4 °C. Appropriate primary antibodies were adopted to analyze immunoprecipitated complexes by western blot, after washing the complexes three times with supplemented RIPA buffer.

#### Tumor xenograft model

BALB/c nude mice (*n* = 20, 5-week-old) were randomly divided into four groups (*n* = 5 per group). RARRES1 overexpressing plasmid- and corresponding control-transfected Huh7 cells were subcutaneously injected into 10 mice, 5 of which were simultaneously intraperitoneally injected with lenvatinib (5 mg/kg) or saline as a control. Tumor volumes were measured using a caliper at 7, 14, 21, 28, and 35 days, and the sensitivity of nude mice to lenvatinib observed. Then, mice were sacrificed and tumors weighed. The Animal Care and Use Committee of The First Affiliated Hospital of Shanxi Medical University approved all experimental procedures.

### Statistical analysis

All data are presented as mean ± standard deviation and were analyzed using Graphpad 9.0. Quantitative variables were analyzed by Student t-test or One-way ANOVA. *P* < 0.05 was considered statistically significant.

### Electronic supplementary material

Below is the link to the electronic supplementary material.

Supplementary Material 1

## Data Availability

The datasets generated and/or analyzed during the current study available from the corresponding author on reasonable request.

## Abbreviations

HCC: Hepatocellular carcinoma
DEGs: Differentially expressed genes
RARRES1: RAR responder protein 1
SPINK2: Serine protease inhibitor Kazal-type 2
TKi: Tyrosine kinase inhibitors
EMT: Epithelial-mesenchymal transition
CCK-8: Cell Counting Kit-8
CO-IP: Co-immunoprecipitation
