# Supplementary material for: RARRES1 inhibits hepatocellular carcinoma progression and increases its sensitivity to lenvatinib through interaction with SPINK2
Source: Biol Direct. 2024 Feb 23;19:15. doi: 10.1186/s13062-024-00459-0 (PMC10885466; doi:10.1186/s13062-024-00459-0)
Supplement: Supplementary file 1 — Supplementary Material 1 [file 13062_2024_459_MOESM1_ESM.docx]

Supplementary Data

Supplementary Table 1. Information on HCC patient samples

| Patients | Age | Gender | TNM stage | HBV | HCV |
| --- | --- | --- | --- | --- | --- |
| 1 | 66 | Male | II | Positive | Positive |
| 2 | 54 | Male | III | Positive | Negative |
| 3 | 78 | Male | III | Positive | Negative |
| 4 | 70 | Female | I | Negative | Negative |
| 5 | 56 | Female | IV | Negative | Negative |
| 6 | 58 | Male | II | Positive | Negative |
| 7 | 62 | Female | II | Positive | Negative |
| 8 | 61 | Male | III | Positive | Negative |
| 9 | 64 | Male | II | Positive | Negative |
| 10 | 62 | Female | I | Positive | Negative |
| 11 | 54 | Male | III | Negative | Positive |
| 12 | 67 | Female | III | Positive | Negative |
